# Paper-Based Flexible Antenna for Wearable Telemedicine Applications at 2.4 GHz ISM Band

**DOI:** 10.3390/s18124214

**Published:** 2018-12-01

**Authors:** Md. Amanath Ullah, Mohammad Tariqul Islam, Touhidul Alam, Farhad Bin Ashraf

**Affiliations:** Centre of Advanced Electronic and Communication Engineering, Faculty of Engineering and Built Environment, Universiti Kebangsaan Malaysia, Bangi, Selangor 43600, Malaysia; touhid13@siswa.ukm.edu.my (T.A.); farhadbinashraf@siswa.ukm.edu.my (F.B.A.)

**Keywords:** flexible antenna, paper antenna, telemedicine, wearable communication, small antenna, paper substrate

## Abstract

This paper demonstrates the performance of a potential design of a paper substrate-based flexible antenna for intrabody telemedicine systems in the 2.4 GHz industrial, scientific, and medical radio (ISM) bands. The antenna was fabricated using 0.54 mm thick flexible photo paper and 0.03 mm copper strips as radiating elements. Design and performance analyses of the antenna were performed using Computer Simulation Technology (CST) Microwave Studio software. The antenna performances were investigated based on the reflection coefficient in normal and bent conditions. The total dimensions of the proposed antenna are 40 × 35 × 0.6 mm^3^. The antenna operates at 2.33–2.53 GHz in the normal condition. More than an 8% fractional bandwidth is expressed by the antenna. Computational analysis was performed at different flexible curvatures by bending the antenna. The minimum fractional bandwidth deviation is 5.04% and the maximum is 24.97%. Moreover, it was mounted on a homogeneous phantom muscle and a four-layer human tissue phantom. Up to a 70% radiation efficiency with a 2 dB gain was achieved by the antenna. Finally, the performance of the antenna with a homogeneous phantom muscle was measured and found reliable for wearable telemedicine applications.

## 1. Introduction

Wearable communication technologies can offer promising solutions in biomedical, consumer electronics, military, and smart home applications. Zimmerman was the very first to discuss body-centric wireless communication systems [1]. The operating frequency of his prototype device was 330 KHz. Since then, body-centric communication systems’ operating frequencies have undergone various changes. In addition to the ultrahigh frequency (UHF) bands that are used for intrabody telemedicine applications, 2.4 GHz is used extensively as an industrial, scientific, and medical (ISM) band [2,3]. Low-profile and lightweight antennas are usually preferred for such applications. However, the height or thickness of the antenna can occasionally be an issue for on-body applications. Moreover, thick antennas lack flexibility; even though they might have flexible features, they can become fragile. To address this problem, flexible paper substrate-based antennas can be utilized since paper is light in weight but has a suitable flexibility limit, depending on the thickness of the substrate. According to the literature, paper-based [4], inkjet-printed antennas have been used for wearable applications [5,6].

Although paper-based, inkjet-printed antennas are cost-effective and easy to manufacture, the conductive ink deteriorates over time, open-circuit phenomena may arise, and the ink is sensitive to heat. In addition, textile antennas can be somewhat flexible [7], but they can also be quite costly, depending on the type of textile. In addition to these types of antennas, other antennas can be found in the literature on wearable communication: for instance, an artificial magnetic conductor (AMC)-backed wearable antenna was described in [8,9]. The antenna used two layers of AMC and Styrofoam between the radiating element and the ground. These layers gave the antenna a total thickness of 9.5 mm, which is quite high.

This paper proposes a new design concept of a paper-based antenna to overcome the shortcomings of other paper-based antennas. The proposed design exploits the lightweight and flexible properties of paper for its application in telemedicine and wearable smart electronic devices for health monitoring. The proposed antenna operates at 2.33–2.53 GHz and resonates at 2.43 GHz. Photo paper was used to construct the 0.54 mm thick substrate, which provides a balance between flexibility and stability in the antenna. A slotted microstrip feed line was used to excite the antenna. The total electrical dimensions of the antenna are 0.31λ × 0.27λ × 0.004λ. First, the antenna was simulated and measured in the normal straight condition. Then, the antenna was measured in the bent condition. In both conditions, the performance is stable in the desired 2.4 GHz ISM band. Then, to validate the flexibility performance of the antenna, computation analysis was performed while bending the antenna into side-by-side and upside-down orientations at different curvature radii. Moreover, a homogeneous phantom muscle and a four-layered human tissue model were mounted with the antenna for computational performance analysis. Finally, the antenna was mounted on a homogeneous phantom muscle, and the reflection coefficient and radiation patterns were studied.

## 2. Design and Methodology

**Figure 1:**
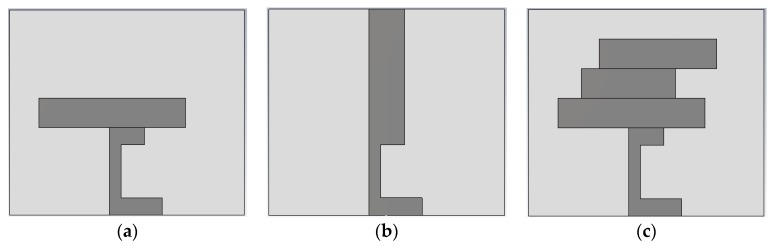
(**a**) Design 1; (**b**) Design 2; (**c**) proposed design.

**Figure 2:**
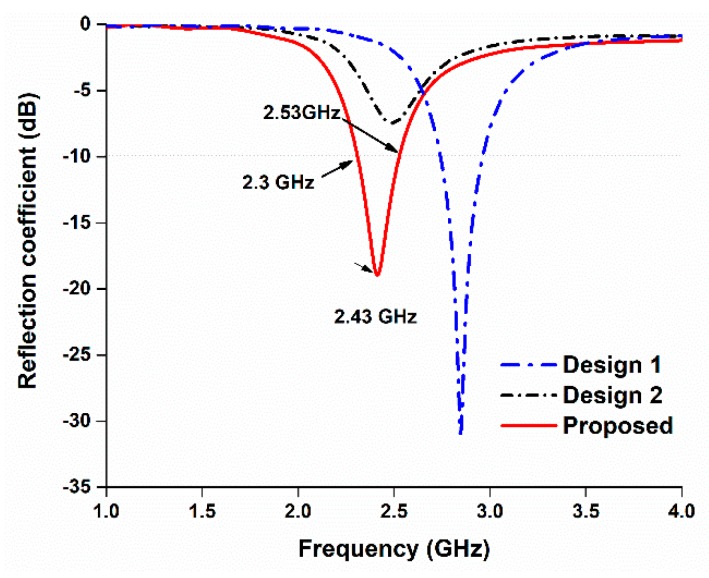
Reflection coefficients of the antenna.

**Figure 3:**
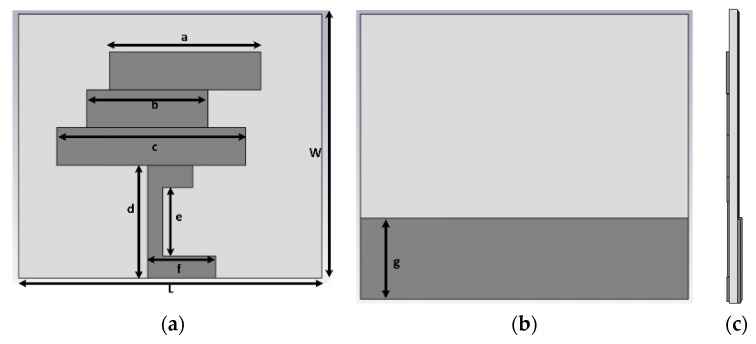
Design configuration of the proposed antenna: (**a**) front view; (**b**) back view; and (**c**) cross-sectional view.

The design and computational analysis of the antenna were performed using Computer Simulation Technology (CST) Microwave Studio software. The primary design process of the antenna started with a basic microstrip-fed antenna, and modification of the radiating patch led to the proposed design. Three designs were simulated to obtain the final design, as depicted in Figure 1. In the proposed design, the length of the microstrip feed is 15 mm and the length and width of the substrate paper material are 40 mm and 35 mm, respectively, providing the antenna with a low profile. Further, 0.54 mm thick photo paper was used as the substrate, and 0.03 mm thick copper strips were used as radiating elements. A partial grounding technique was utilized to obtain a satisfactory operating bandwidth. The corresponding reflection coefficients of the computed paper-based antennas are shown in Figure 2, and the final design of the proposed antenna is illustrated in Figure 3.

**Figure 4:**
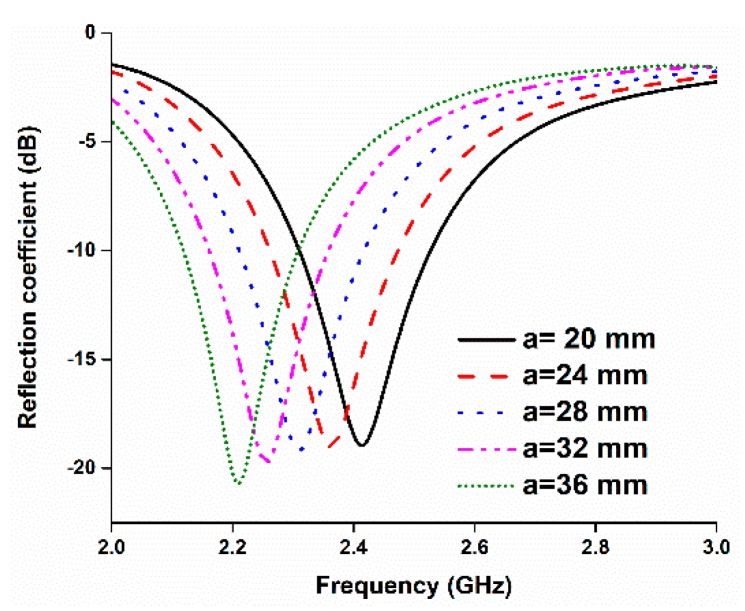
Variation in the reflection coefficient by changing the length of ‘a’.

The length of the upper copper strip has a great impact on the resonant frequency. A parametric study was conducted to determine the optimum length of the upper radiating element. The results are shown in Figure 4. Increasing the length of ‘a’ results in a lower-frequency resonance. Since the length of ‘a’ has a great impact on the resonant frequency, varying the length from 20 to 36 mm shifted the resonant frequency from 2.2 to 2.42 GHz, approximately. The other parameters were obtained through a parametric study, and the optimized design parameters are listed in Table 1.

**Figure 5:**
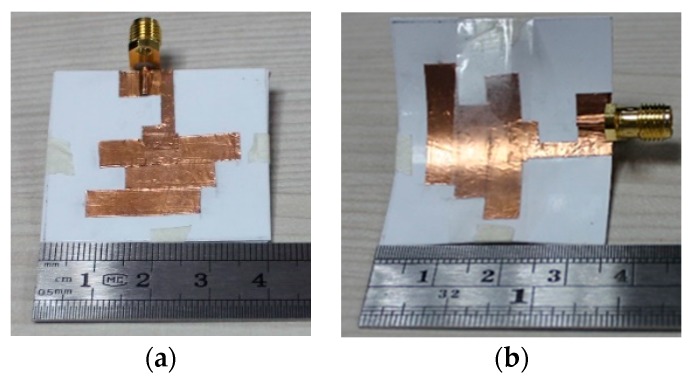
Fabricated antenna prototype in the (**a**) normal condition; (**b**) bent condition.

The prototype was fabricated with commercially available photo paper and a metallic copper strip. The reflection coefficient of the proposed antenna was measured using a performance network analyzer (N5227A) that was calibrated using the Electronic Calibration Module (N4694-60001). Figure 5 represents the fabricated prototype and reflection coefficient measurement of the proposed antenna. The antenna far-field characteristics were investigated using a Satimo near-field measurement system.

**Figure 6:**
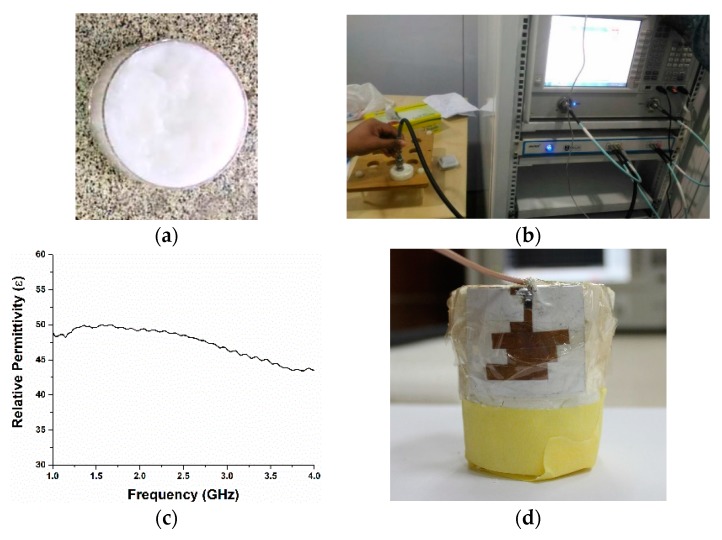
(**a**) Sample material; (**b**) sample material during measurement; (**c**) dielectric property of the muscle phantom; (**d**) antenna mounted with the muscle phantom.

Moreover, a homogeneous phantom muscle was also made to verify the simulated reflection coefficient of the proposed antenna with the phantom muscle, as shown in Figure 6. The homogeneous phantom muscle was fabricated using the methodology described in [10]. The dielectric constant of the fabricated muscle phantom was measured in the Centre for Communication Engineering Studies Lab, Universiti Teknologi MARA (UiTM), Malaysia. The dielectric characteristics of the phantom can be seen in Figure 6b,c.

## 3. Results and Discussion

**Figure 7:**
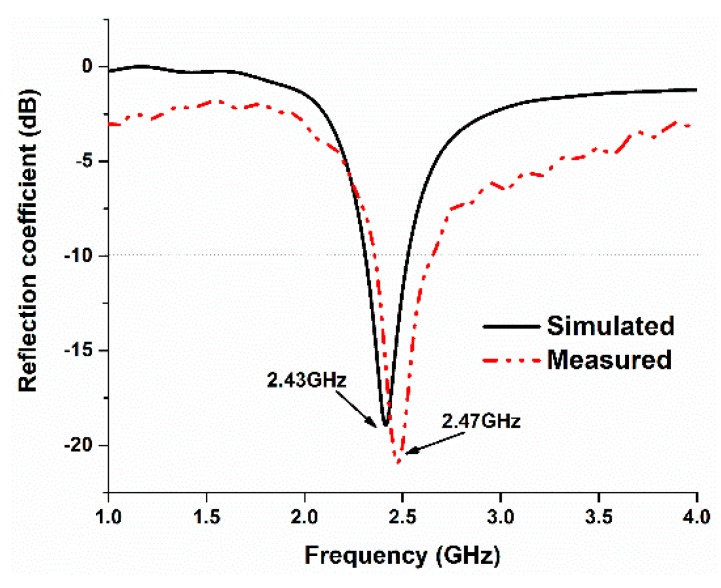
Simulated and measured reflection coefficients of the proposed antenna.

The simulated and measured reflection coefficients of the proposed antenna were analyzed, and the results are illustrated in Figure 7. There is a slight frequency shift in the measured results, which might be due to fabrication and measurement tolerances. In the simulated result, the resonance frequency is 2.43 GHz, and, in the measured result, the resonant frequency is found to be 2.47, which seems to be in good harmony with the computational result.

**Figure 8:**
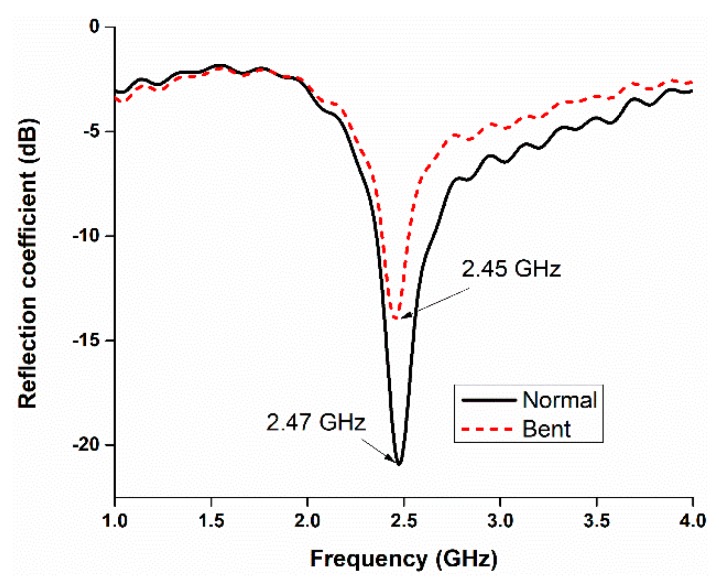
Reflection coefficients during normal and bent conditions.

The reflection coefficient performance of the proposed antenna in the normal and bent conditions was also investigated. It turns out that the antenna shows a stable performance in the bent condition, which authenticates its flexible operation, depicted in Figure 8. There is a slight deviation in the resonant frequency in the bent condition. Bending the antenna yields a resonant frequency that is lower than that in the normal condition by approximately 20 MHz.

**Figure 9:**
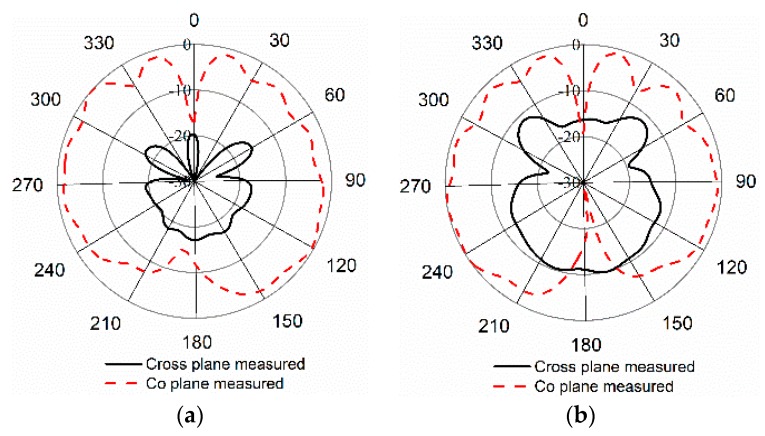
Normalized radiation patterns of the proposed antenna at (**a**) Phi 0; (**b**) Phi 90.

The antenna’s far-field properties were investigated using a Satimo near-field measurement system. In Figure 9, the normalized radiation patterns are depicted for Phi 0 and Phi 90 degrees. One can observe that the antenna shows bidirectional radiation patterns for both planes.

## 4. Bending Deformation Study

**Figure 10:**
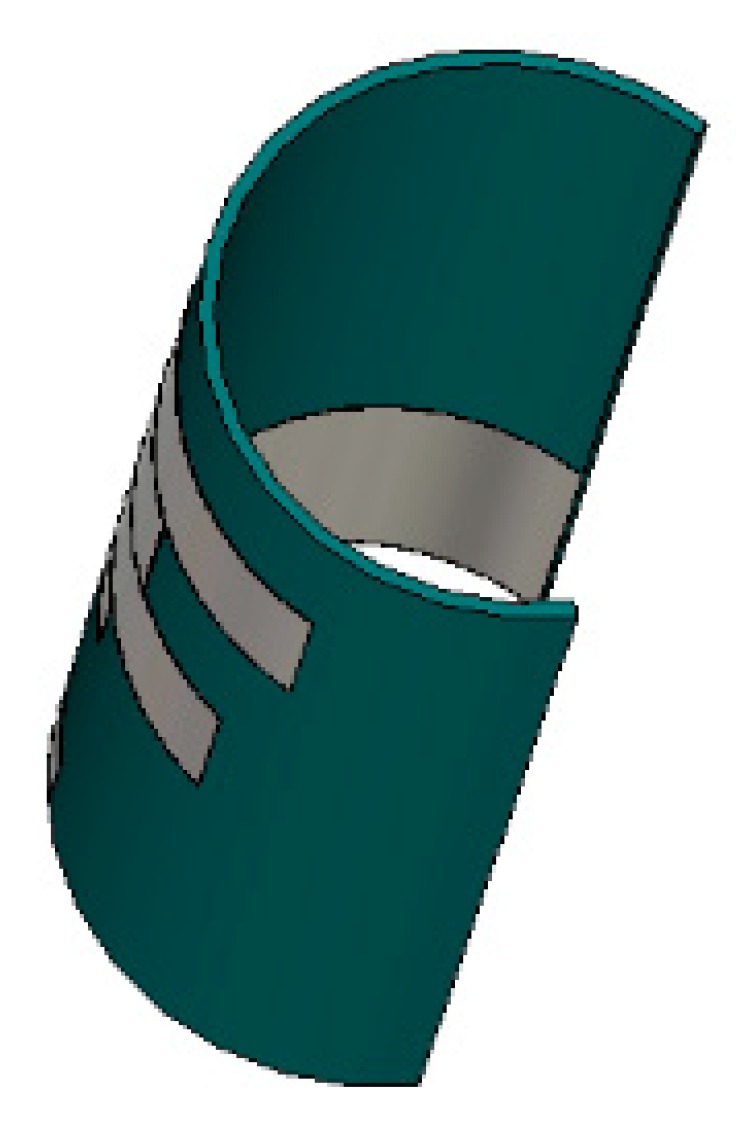
Proposed antenna in a bending condition (side-by-side).

**Figure 11:**
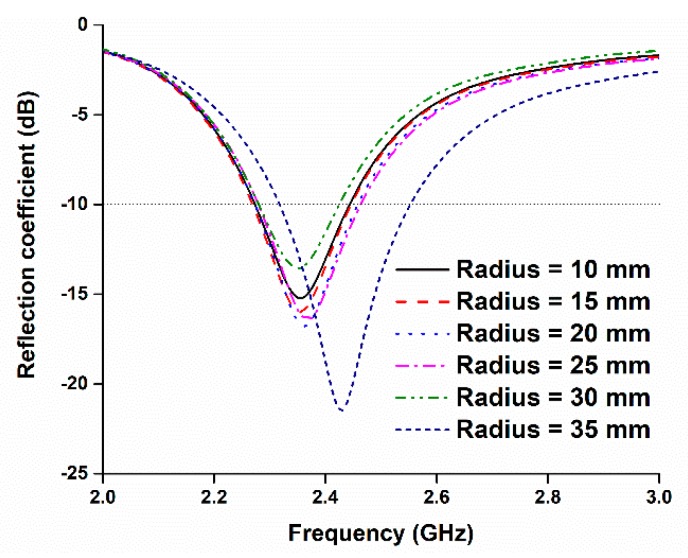
Reflection coefficients of the proposed antenna for different bending (side-by-side) radii.

The performance of the antenna in the side-by-side bending condition at different bending radii was also investigated. Figure 10 depicts the perspective view of the antenna at different bending radii, and Figure 11 shows the reflection coefficients at the corresponding values of the radii. It is evident that the reflection coefficient at the desired band can be found at all the radii when the prototype is bent in a side-by-side orientation. During the side-by-side bending condition, 10, 15, 20, 25, 30, and 35 mm curvature radii exhibit operating bands at 2.27–2.44, 2.26–2.45, 2.27–2.46, 2.28–2.46, 2.27–2.42, and 2.32–2.55 GHz. The maximum bandwidth of 230 MHz is found when the radius is 35 mm.

**Figure 12:**
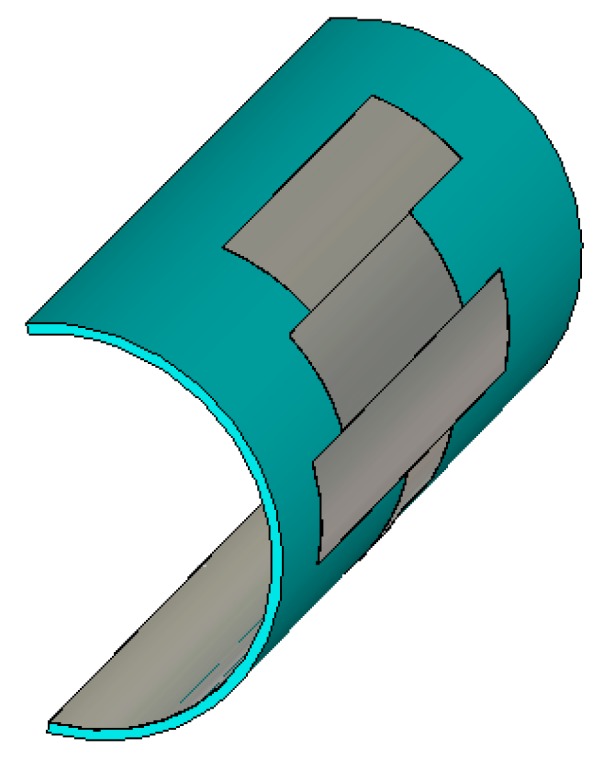
Proposed antenna in a bending condition (upside-down).

**Figure 13:**
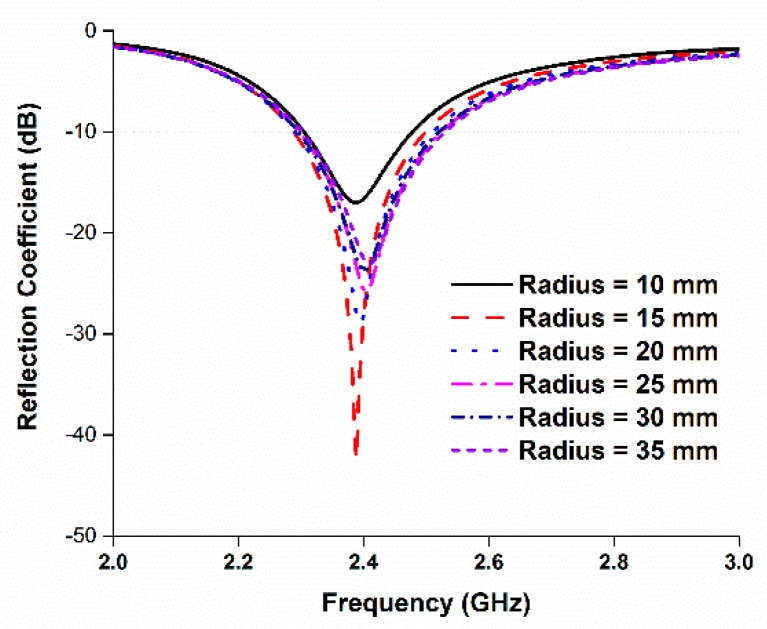
Reflection coefficients of the proposed antenna for different bending (upside-down) radii.

Then, the performance of the antenna was studied during the upside-down condition at different radii, as depicted in Figure 12. The resulting reflection coefficients are depicted in Figure 13. In this case, like in the side-by-side bending condition, the reflection coefficients remain consistent during the upside-down bending condition. Most of the resonant frequencies remain in good harmony at different radii. At a curvature radius of 10 mm, the reflection coefficient is −17.20 dB at 2.38 GHz, which is in the desired 2.30–2.48 GHz ISM band. When the radius is 35 mm, the operating band is found at 2.30–2.53 GHz. The minimum bandwidth of 180 MHz is found at a 10 mm curvature radius, and the maximum bandwidth of 230 MHz is found at a 35 mm curvature radius. 

From Figure 11 and Figure 13, it can be seen that the antenna reflection coefficient remains adequately consistent with various bending radii, and the reflection coefficient can still be found within the desired band. However, a slight change in frequency can be observed for different bending radii. A change in resonant length occurs due to variation in the diameter of bending radii, which impacts the operating frequency [11]. Table 2 represents all the data related to the antenna’s bending performance. The minimum operating band is found to be 170 MHz with a fractional bandwidth of 7.08%. The maximum operating bandwidth is 230 MHz with a fractional bandwidth of 9.58%.

## 5. Antenna Performance with Phantom

**Figure 14:**
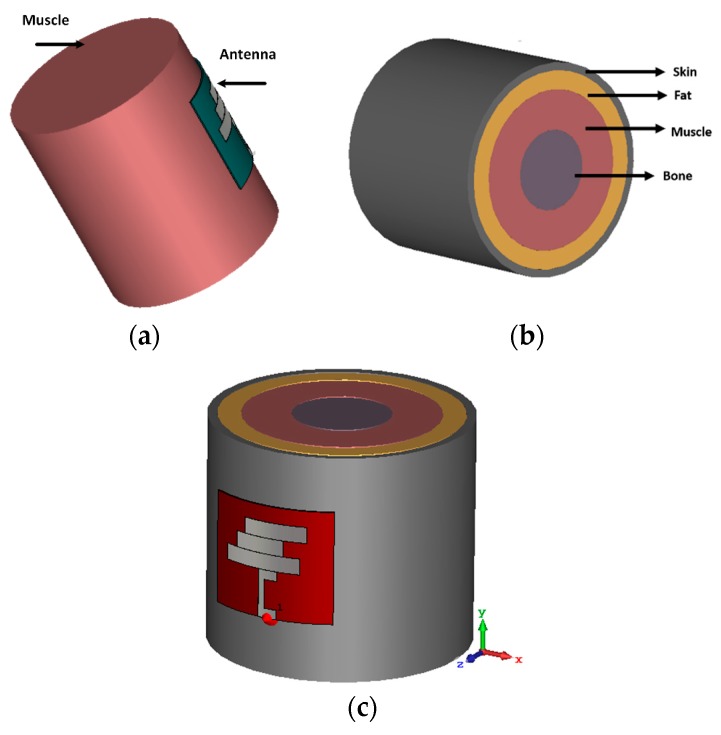
(**a**) Proposed antenna with homogeneous muscle phantom; (**b**) cross-view of the four-layered human tissue phantom; (**c**) antenna with human tissue.

A homogeneous phantom muscle was mounted with the antenna to investigate the antenna’s performance. The simulation setup is shown in Figure 14. The cylindrical phantom shown in Figure 14a was created in CST Microwave Studio using the muscle material available in the material library. This phantom muscle has a dielectric constant of 52. Then, the performance of the antenna attached to a human tissue model with four layers was investigated. The model consists of skin, fat, muscle, and bone, as shown in Figure 14b. The dielectric constants of the modeled skin, fat, muscle, and bone are approximately 37, 5.3, 52, and 11.4, respectively. The thicknesses of the skin, fat, muscle, and bone layers are 3, 7, 15, and 30 mm, respectively. Even though a partial ground plane is used in the antenna, the reflection coefficient remains in good agreement with the reflection coefficient of the antenna in free space.

**Figure 15:**
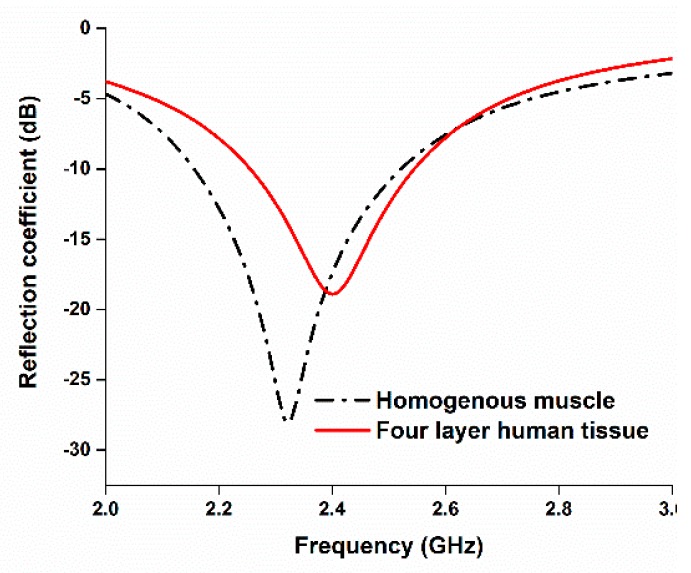
Reflection coefficients of the antenna with homogeneous muscle phantom and four-layer human tissue.

**Figure 16:**
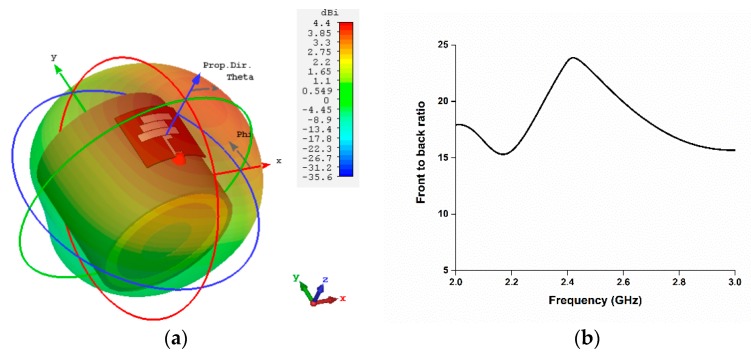
(**a**) Directivity and (**b**) front-to-back ratio of the proposed antenna with human tissue.

The reflection coefficient performance of the antenna with the homogeneous phantom muscle and four-layered human tissue is illustrated in Figure 15. To analyze the directivity of the proposed antenna while mounted on the human tissue, Figure 16 represents the 3D directivity of the antenna. The main direction of propagation is outward from the antenna, which is good for using with the human body.

**Figure 17:**
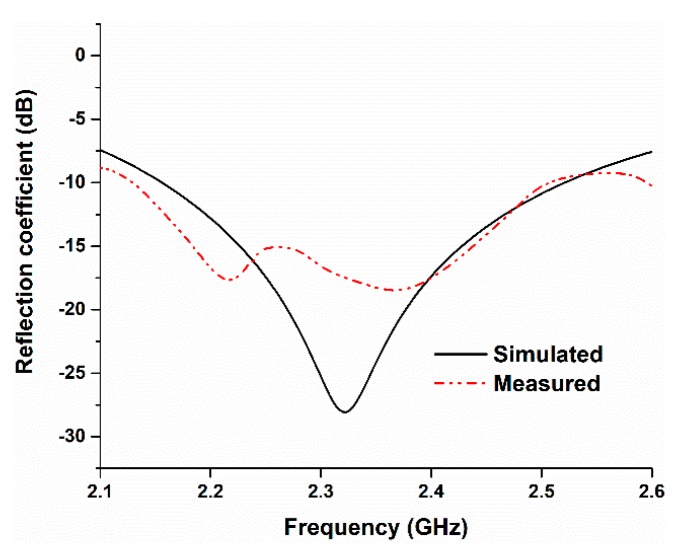
Reflection coefficients of the proposed antenna with the muscle phantom.

**Figure 18:**
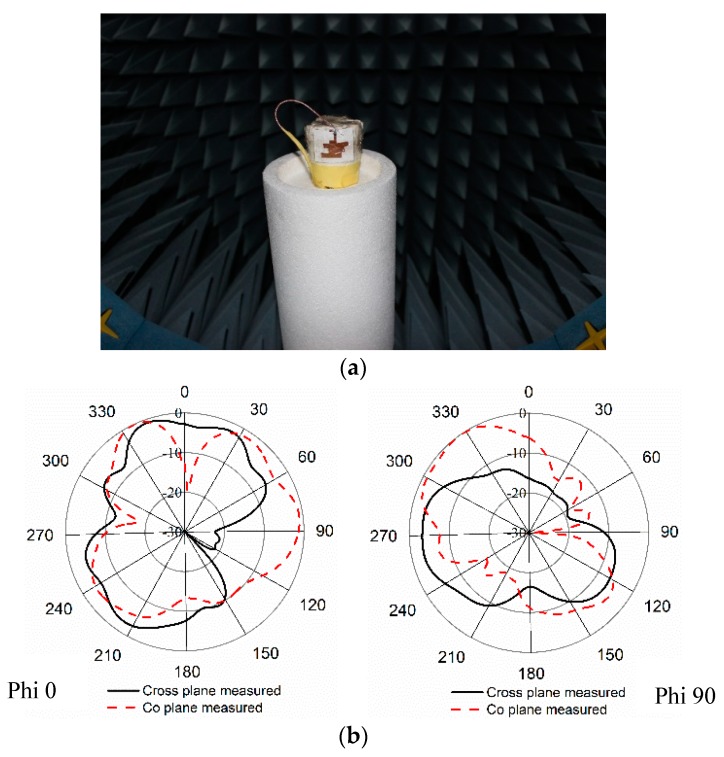
(**a**) Radiation pattern measurement of the proposed paper-based antenna with phantom muscle; (**b**) radiation patterns of the antenna mounted on a muscle phantom at Phi 0 and at Phi 90.

There were noticeable variations in the reflection coefficient between the measured and simulated results with the homogeneous phantom muscle, as shown in Figure 17. It is clear, however, that the bandwidths are in good agreement. One reason for the slight mismatch might be the difference in the dielectric properties of the computational and realistic phantoms. Some other factors could increase the losses of the coupling medium, and the measurement tolerance could impact the results. The radiation patterns of the antenna with the muscle are shown in Figure 18b. The radiation patterns with the phantom muscle remain stable to a satisfactory degree. The overall performance of the antenna seems to be in good harmony when in free space and with the phantom muscle.

**Figure 19:**
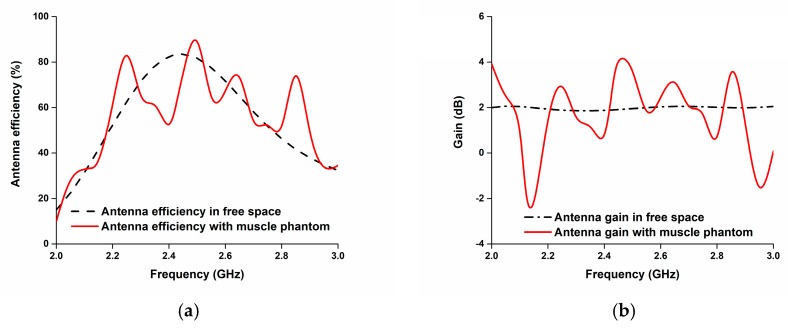
(**a**) Efficiency and (**b**) gain of the proposed antenna.

Figure 19 presents the total efficiency and gain of the proposed paper-based flexible antenna. It achieves up to an 83% efficiency in free space and up to a 70% efficiency when mounted on the phantom at 2.45 GHz; it also obtains more than a 2 dB gain in the operating band.

Achieving a good performance from a compact antenna is a challenge when the application is wearable communication. From Table 3, it can be seen that the proposed antenna is sufficiently compact with a minimum thickness of only 0.6 mm, which makes the antenna a perfect candidate for wearable telemedicine applications.

## 6. Conclusions

This paper illustrates a new design and performance analysis of a paper substrate-based flexible antenna. The prototype was fabricated for wearable telemedicine applications in the 2.4 GHz band. This paper presents the reflection coefficients for both normal and flexible conditions. The antenna is only 0.6 mm thick and can serve as a wearable system with a satisfactory flexibility range. In addition, the performance of the antenna with human tissue was investigated. The results for the reflection coefficient and radiation patterns are found to be satisfactory for wearable telemedicine applications.

## Figures and Tables

**Table 1:** Design parameters of the proposed antenna.

Parameters	Value (mm)	Parameters	Value (mm)
L	40	W	35
a	20	b	16
c	25	d	15
e	12	f	5

**Table 2:** Bending performance data.

Bending Condition	Curvature Radius	Bandwidth	Fractional Bandwidth	Deviation
Normal	None	200 MHz (2.33–2.53 GHz)	8.33%	-
Side-by-side	10	170 MHz (2.27–2.44 GHz)	7.08%	15.00%
	15	190 MHz (2.26–2.45 GHz)	7.91%	5.04%
	20	190 MHz (2.27–2.46 GHz)	7.91%	5.04%
	25	180 MHz (2.28–2.46 GHz)	7.50%	9.96%
	30	150 MHz (2.27–2.42 GHz)	6.25%	24.97%
	35	230 MHz (2.32–2.55 GHz)	9.58%	−15.00%
Upside-down	10	180 MHz (2.30–2.48 GHz)	7.50%	9.96%
	15	210 MHz (2.29–2.50 GHz)	8.75%	−5.04%
	20	220 MHz (2.29–2.51 GHz)	9.17%	−10.08%
	25	220 MHz (2.30–2.52 GHz)	9.17%	−10.08%
	30	220 MHz (2.29–2.51 GHz)	9.17%	−10.08%
	35	230 MHz (2.30–2.53 GHz)	9.58%	−15.00%

**Table 3:** Comparison of different wearable antenna.

Reference Antenna	Type of Wearable Antenna Material	Operating Band	Size	Thickness
[4]	Paper	1.4–1.65 GHz	90 × 74.8 mm^2^	n/a
[12]	FR4	2.40–2.48 GHz	38 × 38 mm^2^	3 mm
[13]	Resin-coated paper	0.8–1.1 GHz	60 × 60 mm^2^	5.14 mm
[14]	Jeans	2.4–2.5 GHz	120 × 120 mm^2^	3.5 mm
Proposed	Photo paper	2.33–2.53	40 × 35 mm^2^	0.6 mm
